# Geographical Area and Life History Traits Influence Diet in an Arctic Marine Predator

**DOI:** 10.1371/journal.pone.0155980

**Published:** 2016-05-19

**Authors:** Sabrina Tartu, Sophie Bourgeon, Jon Aars, Magnus Andersen, Dorothee Ehrich, Gregory W. Thiemann, Jeffrey M. Welker, Heli Routti

**Affiliations:** 1 Norwegian Polar Institute, Fram Centre, Tromsø, Norway; 2 UiT-The Arctic University of Norway, Department of Arctic and Marine Biology, Tromsø, Norway; 3 York University, Faculty of Environmental Studies, Toronto, ON, Canada; 4 Department of Biological Sciences, University of Alaska Anchorage, Anchorage, Alaska, United States of America; 5 University Center in Svalbard, Longyearbyen, Svalbard, Norway; Université du Québec à Rimouski, CANADA

## Abstract

Global changes are thought to affect most Arctic species, yet some populations are more at risk. Today, the Barents Sea ecoregion is suffering the strongest sea ice retreat ever measured; and these changes are suspected to modify food access and thus diet of several species. Biochemical diet tracers enable investigation of diet in species such as polar bears (*Ursus maritimus*). We examined individual diet variation of female polar bears in Svalbard, Norway, and related it to year, season (spring and autumn), sampling area and breeding status (solitary, with cubs of the year or yearlings). Sampling areas were split according to their ice cover: North-West (less sea ice cover), South-East (larger amplitude in sea ice extent) and North-East/South-West (NESW) as bears from that zone are more mobile among all regions of Svalbard. We measured fatty acid (FA) composition in adipose tissue and carbon (δ^13^C) and nitrogen (δ^15^N) stable isotopes in plasma and red blood cells. Females feeding in the North-West area had lower δ^15^N values than those from the NESW. In South-East females, δ^13^C values were lower in autumn compared to spring and females seemed less selective in their diet as depicted by large variances in stable isotope values. Considering the differences in FA composition and stable isotope values, we suggest that females from the North-West and South-East could ingest a higher proportion of avian prey. With regard to breeding status, solitary females had higher δ^15^N values and smaller variance in their stable isotopic values than females with cubs, suggesting that solitary females were more selective and prey on higher trophic level species (i.e. seals). Overall, our results indicate that prey availability for Svalbard polar bears varies according to geographical area and prey selectivity differs according to breeding status. Our findings suggest that complex changes in sea ice and prey availability will interact to affect Svalbard polar bear feeding patterns and associated nutrition.

## Introduction

The Arctic marine environment is characterized by a dynamic sea ice platform that varies in its seasonal and spatial distribution, as well as its form and type [1]. Over the last decades, the Arctic has experienced the greatest warming ever measured and sea ice extent has undergone dramatic reductions [2–5]. The Arctic marine biota relies on sea ice as a physical platform to breed and to feed and is thus vulnerable to changes in ice characteristics [6]. With ongoing climate warming and sea ice reduction, diet composition of pagophilic marine mammals is expected to change [7–9]. Yet, studying the diet of sea ice dependent species such as polar bears (*Ursus maritimus*) or seal species can be a challenge given their low density populations [10,11].

Diet habits of polar bears have been well documented, they are the most carnivorous ursid species and they mainly prey upon ringed and bearded seals (*Pusa hispida* and *Erignathus barbatus*, respectively) [12–14]. Nevertheless, they are also opportunistic and scavenger feeders [14]. This includes seabirds, waterfowl, eggs, reindeer (*Rangifer tarandus*), fish and whale carcasses [15–24]. With ongoing reductions in sea ice availability, terrestrial feeding may become more frequent [20,22,24–27] although the energetic and ultimate ecological value of these foods for polar bears remains a matter of debate [24,27].

Among subpopulations that received a high monitoring intensity such as southern Beaufort Sea, western Hudson Bay and the Barents Sea’s subpopulations [28], the diet of polar bears from Svalbard, has been seldom investigated. Only a handful of studies have described diet through opportunistic observation of stomach contents, seal kills, scat analysis or fatty acid (henceforth ‘FA’) composition [12,19,29,30]. The Barents Sea area is experiencing the fastest loss of sea ice recorded throughout the Arctic, with a reduction of 41.8 ± 7.1 ice-free days per decade [5]. There is however, a lack of information on population trends of marine mammals inhabiting this region, including polar bears [5]. Polar bears from the Barents Sea subpopulation are suspected to be particularly vulnerable to the consequences of climate change, which will be primarily mediated by the access to sea ice habitat, and thus reduced foraging opportunities [31]. Thus, it is crucial to understand better the dietary ecology of Svalbard polar bears.

Life-history traits may explain a significant amount of individual variability in diet choices and habitat use in polar bears [32]. For instance, females undergo a fasting period associated with parturition from about four to eight months, varying with area [33]. Only a few studies have reported differences in habitat use according to breeding status in female polar bears [32,34] but there is to our knowledge no evidence that females with different breeding status feed on different prey. However, we assume that females will have different energetic needs depending on the presence, age and number of offspring, which may influence prey selection. Their dependence on seasonal and variable habitat–sea ice–together with their complex biology makes polar bears an ideal model organism to study how diet may vary depending on environmental conditions and life history status.

Biochemical tracers, for instance FA profiles (i.e. the relative proportion of various FA) or nitrogen and carbon stable isotopes, have been used as proxies to describe the foraging niche and diet composition of free-ranging species [14,35–37]. This occurs because the chemistry of animal tissues reflects to some degree the biochemical properties of their food. Indeed, FA can provide insight into an organism’s diet because dietary FA are predictably incorporated into a consumer’s tissues. The FA composition in adipose tissue can provide an integrated record of dietary intake over a period of weeks to months, and perhaps longer in some species [38]. Moreover, nitrogen stable isotope ratios (^15^N/^14^N expressed as δ^15^N) fractionate or change in a predictable fashion between trophic levels and so reflect trophic position [39]. Carbon isotope ratios (^13^C/^12^C expressed as δ^13^C) remain stable and thus indicate isotopic signatures of sources of primary productivity [39], for example marine vs. terrestrial, pelagic vs. benthic, inshore vs. offshore. Stable isotopes in red blood cells integrate the diet of the last 2–3 months while the turnover of stable isotopes in plasma is in the order of 1–2 weeks [39,40]. Therefore, concurrent analyses of stable isotopes and FA can provide a robust understanding of marine mammal foraging behavior [40]. Additionally, these diet tracers have several advantages compared to other methods to determine diet. For example, they can integrate diet over a longer time window than polar bear kill sites monitoring or scat analyses. Moreover, they require the capture of the individual, therefore diet can be related to other individual information (biometry, status, physiology, etc.) and finally diet tracers are not destructive, on the contrary to studying stomach content.

We investigated individual variation in the diet of female polar bears and related it to environmental parameters and life history. Due to spatio-temporal variation in sea ice extent we expected the diet of bears to differ between spring and fall, possibly reflecting a larger proportion of seals available during the spring. Additionally, we expected diet to differ between areas in Svalbard according to the timing, amplitude and duration of ice cover; which may influence prey availability. Then, we hypothesized that solitary females will have different feeding strategies than females with dependent offspring because of differences in mobility of the latter (e.g. reduced mobility, risk of infanticide) but also because of increased energetic needs for further reproductive events.

## Materials and Methods

### Ethic statement

The National Animal Research Authority (NARA), Norway, approved research and capture protocols (permit number FOTS ID 7278).

### Field sampling

Adult female polar bears (age 4–28 years) from the Barents Sea subpopulation were captured in April and September 2012 and 2013. The 112 samples collected (N = 33 in April 2012, N = 24 in September 2012, N = 29 in April 2013 and N = 26 in September 2013) represented 78 females with 26 animals being captured more than once. Females were sampled opportunistically throughout Svalbard and immobilized by remote injection of tiletamine hydrochloride and zolazepam hydrochloride (Zoletil ® 100; Virbac, France), delivered by a dart fired from a helicopter (Eurocopter AS350 Ecureuil). Blood was collected from the femoral vein using heparinised collecting tubes. We kept samples cool and out of sunlight until centrifuged within 10 h (3500 rpm, 10 minutes). Red blood cells and plasma were frozen and stored at -20°C and subsequently used to assess stable isotope values. Adipose tissue samples were collected using a 8 mm biopsy punch and consisted of a full-layer core from skin to muscle, taken approximately 15 cm lateral to the base of the tail [41]. Tissue samples were stored in liquid nitrogen in the field, and kept at -80°C until analysis. Immobilization and handling procedures followed standard protocols [42,43]. Females were classified in three groups according to their breeding status: solitary (i.e. alone or together with a male in spring), with 1 or 2 cubs of the year (COYs; cubs younger than 1 year old) or with 1 or 2 yearlings (YRLs; cubs aged between 1 and 2 years). Additionally, we reported opportunistic observations of kills and carcasses for the captured females and identified the prey species when possible.

### Sampling areas determination

**Fig 1:**
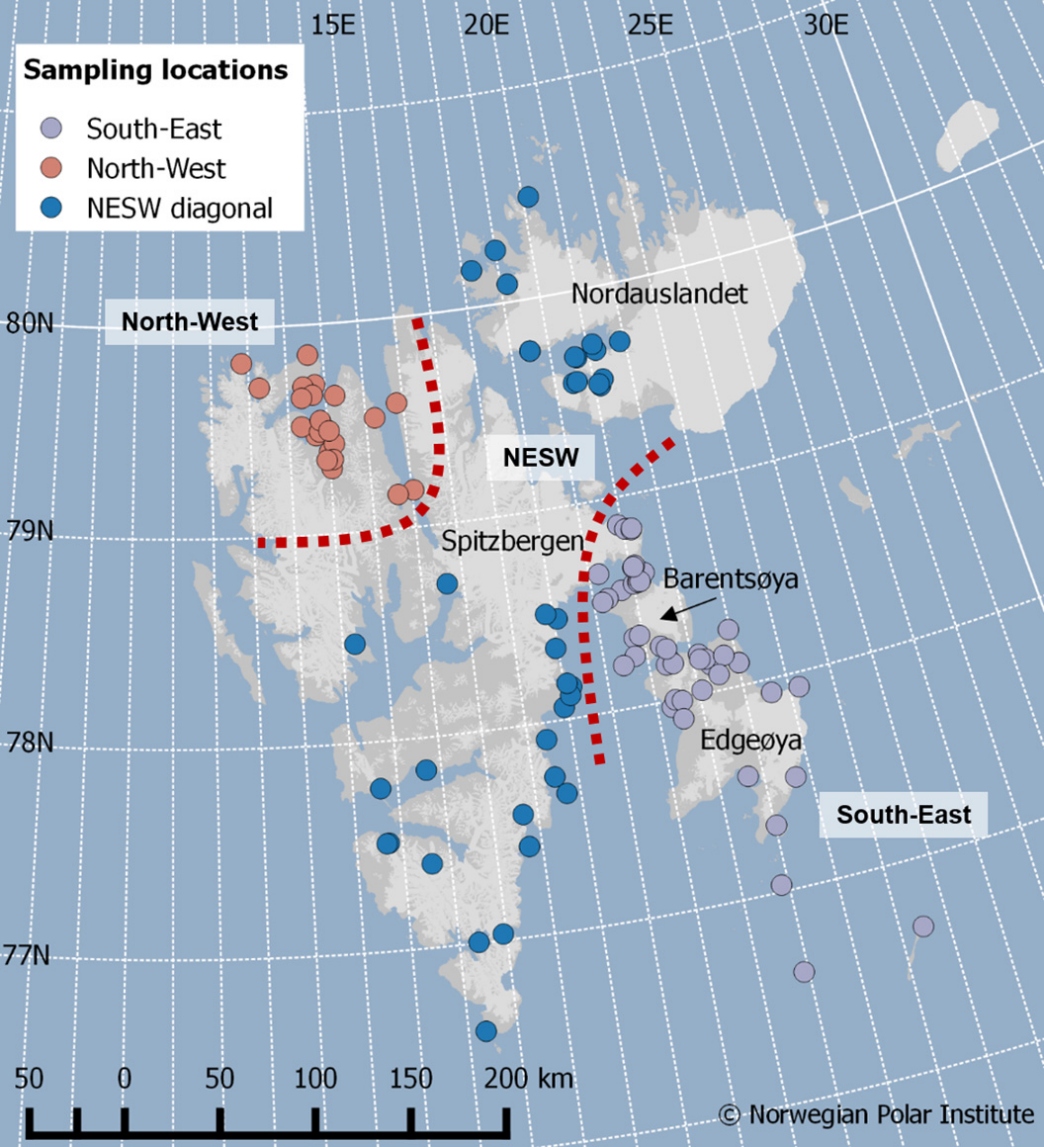
Location of the 112 samples collected from 78 females in Svalbard. Female polar bears were captured in 2012 and 2013. Each circle represents a polar bear sampled for blood and adipose tissue. The three regions are represented by different colors: North-West (orange dots), North-East/South-West diagonal (blue dots) and South-East (grey dots).

Owing to physical and chemical processes, sea ice concentration, the timing of ice availability and the duration of the ice season, are irregular on the different coasts of Svalbard [44–46]. Specifically, the sea ice is less extended and its density lower along the West coast of Svalbard compared to the Eastern coast [44–46]. The South-East area of Svalbard, including the islands Barentsøya and Edgeøya, experiences the largest amplitude of sea ice retreat during summer [44–46]. Consequently, we separated females caught in the North-West from those caught in the South-East area. Bears caught in the remainder of the archipelago, i.e. Nordaustlandet and along the North-East and Southern coasts of Spitsbergen (henceforth the North-East/South-West diagonal: NESW) frequently move among all regions (J. Aars, unpublished data), we therefore pooled them into a third group (Fig 1). Only two females were captured in more than one of these areas; both were caught in the NESW area in spring and recaptured in the South-East area in autumn.

### Stable isotopes and FA determination

The determination methods for FA from adipose tissue [38] and stable isotopes in plasma and red blood cells [14] are detailed in S1 Appendix. We collected blood and adipose tissue from all the captured females. We measured stable isotopes in plasma and red blood cells for 112 samples (78 individuals) and FA data were derived from 83 samples (64 individuals) as in the remainder, fat biopsies were too small to determine FA composition (S1 Table provides an overview of the samples). From 75 FA determined in fat samples, we used 32 FA representing more than 0.2% of total mass % FA in subsequent analyses (S2 Table) [47]. Overall, 33 FA were ≥ 0.2% of total FA. Percentage values for FA were transformed by calculating the log of the ratio of each FA to c18:0 prior to principal component analysis (PCA) [38]. Since the log of 0 cannot be taken, 0 values were replaced with a small constant (0.005%) prior to transformation. We used PCA ordination plots to illustrate the FA patterns according to sampling area, season and breeding status. Ordination plots will order individuals that are characterized by PCA scores, so that individuals with similar PCA scores are near each other and individuals with dissimilar PCA scores are farther from each other. The 32 FAs (without c18:0) used in the present study included i-14:0, c14:0, 14:1n-5, c15:0, c16:0, 16:1n-11, 16:1n-9, 16:1n-7, 16:1n-5, i-17:0, 16:2n-4, c17:0, 18:1n-11, 18:1n-9, 18:1n-7, 18:1n-5, 18:2n-6, 18:3n-4, 18:3n-3, 18:4n-3, 20:1n-11, 20:1n-9, 20:1n-7, 20:2n-6, 20:4n-6, 20:4n-3, 20:5n-3, 22:1n-11, 22:1n-9, 21:5n-3, 22:5n-3 and 22:6n-3.

### Statistical analyses

We conducted statistical analyses using R version 3.2.1 [48]. We ran a PCA on the 32 FA (R package *ade4*, [49]). We generated FA principal components (PCs) for further analysis from the first, second and third axis of the PCA (projected inertia: PC1: 31.6, PC2: 16.7, PC3:12.7%, respectively). The three first axes extracted 61.0% of the total variance of the data cloud. To test for the effects of sampling area, year, season, breeding status and two-way interactions (fixed factors) on FA PCs 1, 2 and 3, δ^13^C, and δ^15^N values, we used generalized linear mixed models (GLMMs; R-package *nlme* version 3.1–121; [50]. We used female identity (FemID) as a random factor to account for the repeated measurements (among seasons and/or years). To select the best GLMMs, we used an information-theoretic approach [51] based on Akaike’s information criterion corrected for small sample size (AICc, R package *AICcmodavg* version 2.0–3, [52]). We tested 15 biologically relevant candidate models based on the variables included (S3 Table). The best model was taken to be the one with the smallest AICc. Yet among models with ΔAICc < 2, we selected the most parsimonious one [51]. We obtained parameter estimates and 95% confidence intervals (CIs) for the selected models. The level of significance was set to α ≤ 0.05. When the interaction term was significant, we disregarded the effects of the main factors on the response variable and we used least squares means method (*lsmeans* function in *Lsmeans* package, [53]) to identify significantly different terms in a biologically relevant frame. For example, if the interaction of year and season is significant, we will not compare bears captured in spring 2012 to those captured in autumn 2013. We will rather check if in 2012 there was an effect of season we do not observe in 2013. With regard to FA, we tested whether some FA, more abundant in certain prey or characteristic of some arctic food chains, would differ according to environmental factors and breeding status. To do so, we selected FA 22:1n-11 as a marker of pelagic prey [54] and 18:1n-9 and c16:0 as markers of bird prey [55,56]. We checked the proportions of FA 22:6n-3, c18 polyunsaturated FA (PUFA: 18:2n-6, 18:4n-3), 20:5n-3, 22:5n-3 and 16:1n:7 to determine whether the diet was based on a diatom food-chain characteristic of harp seals, (*Pagophilus groenlandicus*), or a dinoflagellate and prymnesiophyte (*Phaeocystis pouchetii*) food-chain characteristic of hooded seals, (*Cystophora cristata*) [57,58]. We also focused on FA known to be readily or poorly mobilized during periods of nutritional stress [59]. We selected five FA, which were highly represented in polar bear adipose tissues in this study: FA 22:1n:11, 20:1n-9, 22:1n-9 for the least mobilized FA and FA 16:1n-7 and 20:5n-3 for the readily mobilized FA. We tested differences in FA mass % according to variables selected for PC1, PC2 and PC3 by AICc (Table 1) and least square means method [53]. Finally, to test whether prey selectivity differed according to sampling area, year, season and breeding status we performed Levene variance tests on δ^13^C and δ^15^N values in red blood cells and plasma (*levene*.*test* function in *lawstat* package [60]). We assumed a smaller variance reflects a more specific diet.

**Table 1:** Model selection using the AICc to determine the most parsimonious model explaining variation in stable isotope and fatty acid principal component values of female polar bears sampled in Svalbard in 2012 and 2013.

Response variable	Model	K	AICc	ΔAICc	AICc Weight
Fatty acids PC1	Year + Season + Status + Season × Status + Year × Season	10	418.99	0.00	0.61
	**Season + Status**	6	420.68	1.69	0.26
	Season + Status + Season × Status	8	423.89	4.90	0.05
	Sampling area + Status	7	424.28	5.29	0.04
Fatty acids PC2	**Sampling area + Season**	6	357.99	0.00	0.71
	Season	4	362.01	4.01	0.10
	Season + Status	6	362.57	4.57	0.07
	Sampling area + Season + Sampling area × Season	8	362.60	4.60	0.07
Fatty acids PC3	**Sampling area**	5	333.60	0.00	0.32
	Sampling area + Season + Sampling area × Season	8	334.10	0.50	0.25
	Sampling area + Status	7	334.71	1.11	0.18
	Sampling area + Year	6	335.88	2.28	0.10
δ^13^C values red blood cells	Sampling area + Season	6	227.90	0.00	0.31
	Season	4	228.20	0.30	0.27
	Null model	3	229.60	1.75	0.13
	Year	4	231.10	3.27	0.06
δ^13^C values plasma	**Sampling area + Season + Sampling area × Season**	8	274.80	0.00	0.95
	Sampling area + Season	6	282.10	7.32	0.02
	Sampling area + Year	6	284.00	9.17	0.01
	Year + Season + Status + Season × Status + Year × Season	10	284.60	9.77	0.01
δ^15^N values red blood cells	**Sampling area + Status**	7	328.30	0.00	0.34
	Sampling area + Season	6	330.60	2.27	0.11
	Sampling area	5	330.60	2.28	0.11
	Sampling area + Year	6	331.00	2.69	0.09
δ^15^N values plasma	**Season + Status**	6	377.00	0.00	0.61
	Season + Status + Season × Status	8	379.60	2.63	0.16
	Season	4	380.30	3.31	0.12
	Year + Season + Status + Season × Status + Year × Season	10	381.80	4.83	0.05

All models (linear mixed models) include female identity as a random factor. The most competitive models for each diet tracer are represented and ranked according to their AICc, the selected model (i.e. the most parsimonious among the models with ΔAICc < 2) appears in bold. K indicates the number of parameters, ΔAICc the difference in AICc between each candidate model and the model with the lowest AICc and AIC weight the Akaike weights. The different variables are year (2012, 2013), sampling area (North-West, NESW, South-East), season (Spring, Autumn) and breeding status (solitary, with COYs, with YRLs).

## Results

### Predictors of FA in adipose tissue

**Fig 2:**
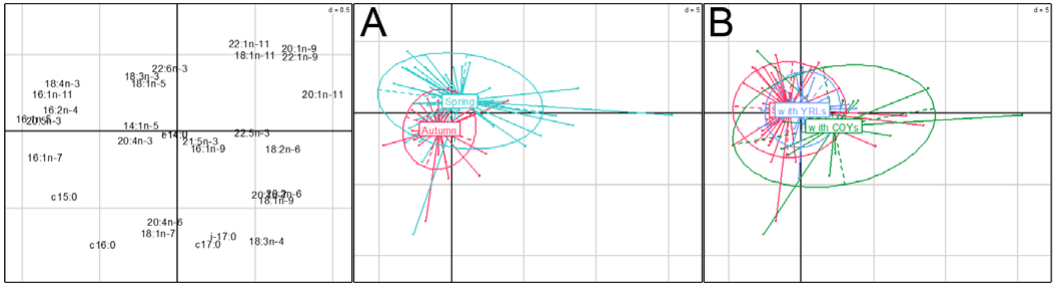
**Ordination plots from fatty acids (FA) principal component analysis (PCA) scores grouped by (A) season and (B) breeding status.** PCA was based on mass percentage of total dietary fatty acids (FAs) relative to FA 18:0, in female polar bears sampled in Svalbard in 2012–2013. The first and second axes explained 31.6% and 16.7% of the total variation, respectively. FA contribution to each axis appears on the left panel. On panels A and B, each dot represents an individual. Individuals with similar FA composition (PCA scores) are near each other and individuals with dissimilar FA composition are farther from each other, the center of the star linking all individuals of a same group represents the centroid of PCA scores for that group.

**Fig 3:**
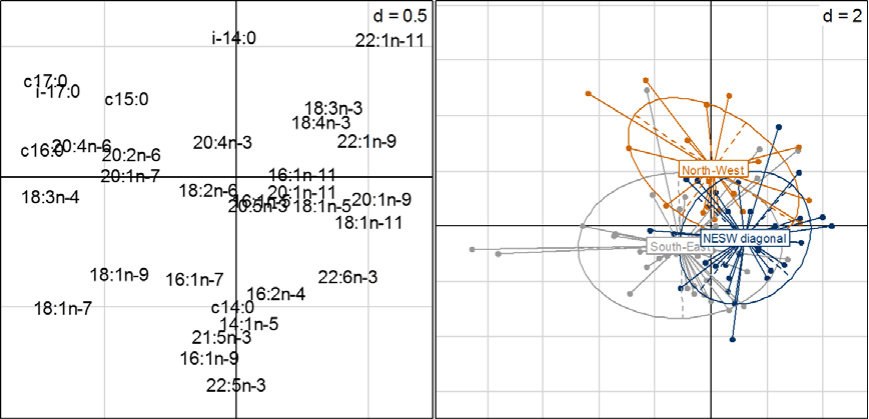
Ordination plot from fatty acids (FA) principal component analysis (PCA) scores grouped by sampling area. PCA was based on mass percentage of total dietary FAs relative to FA 18:0, in female polar bears sampled in Svalbard in 2012–2013. The second and third axes explained 16.7% and 12.7% of the total variation, respectively. See Fig 2 for the interpretation of an ordination plot.

The variables which best predicted FA PC1 value were season and breeding status (Table 2, Fig 2A and 2B). In autumn, PC1 values in female polar bears were lower compared to females caught in spring. PC1 values were less in solitary females compared to that of females with COYs, but they did not differ between solitary females and those with YRLs (Table 2). The best predictors of FA PC2 values were season and sampling area, yet the effect of sampling area was not considered significant as the 95% CI included 0 (Table 2). FA PC2 values were also lower in females caught in autumn compared to those caught in spring (Table 2). Finally, the best predictor of FA PC3 was sampling area (Table 2, Fig 3). Females caught in the NESW diagonal and those caught in the South-East had lower FA PC3 values than females caught in the North-West (Table 2).

**Table 2:** Relationships between fatty acids’ PCA scores, environmental factors and breeding status in female polar bears.

Response	Fatty acids PC1		Fatty acids PC2		Fatty acids PC3	
Selected model	Season + Status		Sampling area + Season		Sampling area	
	Estimate	95% CI	Estimate	95% CI.	Estimate	95% CI
**Fixed Parts**						
Intercept	-0.28	-1.24 – 0.68	0.71	-0.43 – 1.86	2.09	1.29 – 2.89
Autumn	-1.73	-2.91 – -0.54	-1.38	-2.20 – -0.56		
NESW			0.86	-0.49 – 2.21 ^a^	-2.53	-3.53 – -1.52
South-East			-0.87	-2.19 – 0.45 ^a^	-2.79	-3.78 – -1.81
with COYs	3.34	1.73 – 4.95				
with YRLs	1.17	-0.39 – 2.73^a^				
**Random Parts**						
N_FemID_		64		64		64
ICC_FemID_		0.257		0.497		0.045
Observations		83		83		83

Females were sampled from Svalbard area in 2012–2013. Values are parameter estimates and 95% confidence intervals (CI) for the best general linear mixed models according to AICc selection. All models (linear mixed models) include Female identity (FemID) as a random factor, sampling area, season and breeding status as fixed factors. Parameter estimates represent contrasts to the reference level which was spring, north-west, solitary female in 2012. For the random parts, N is the number of individuals, ICC is the intraclass correlation coefficient and Observations the number of samples.

^a^This effect is not supported because the 95% CI included zero.

**Fig 4:**
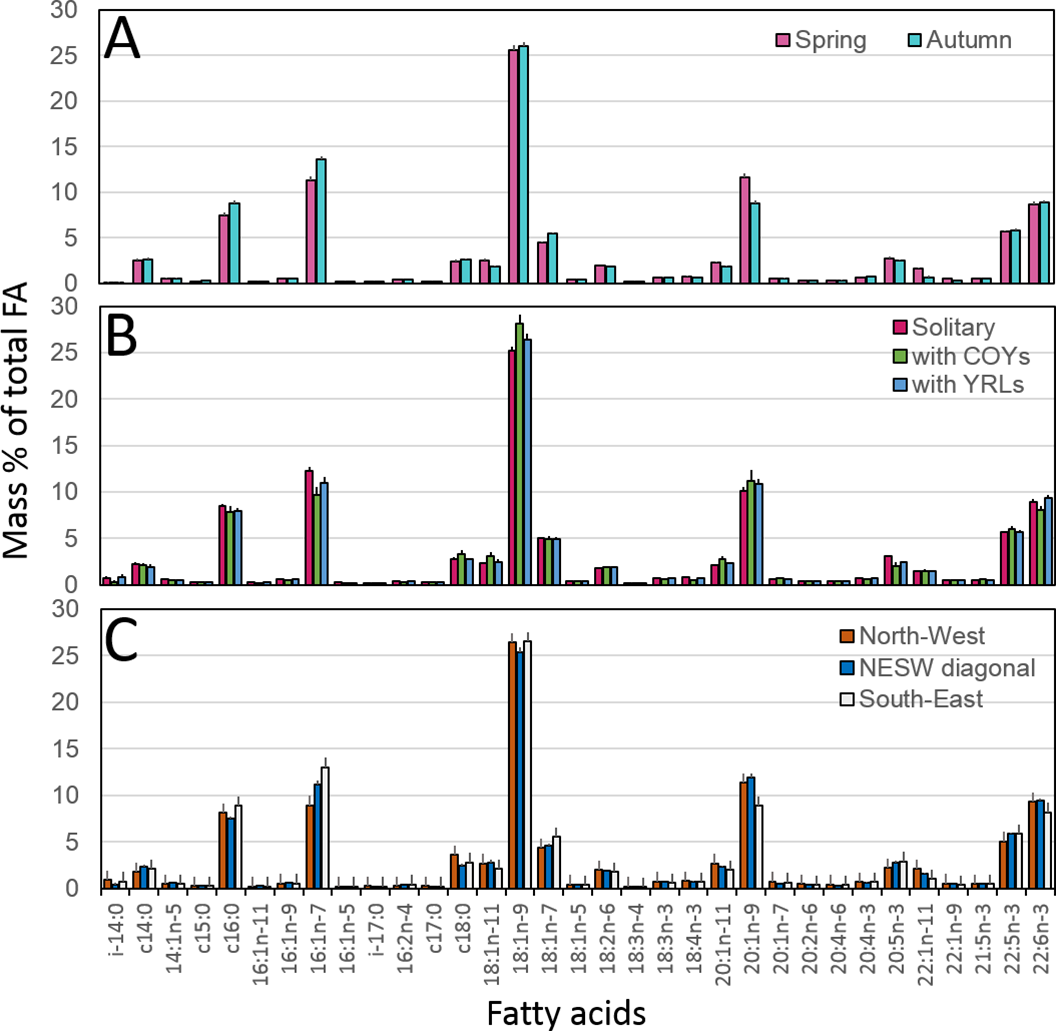
**Median mass % of 33 fatty acids (FA) according to A) season, B) breeding status and C) sampling area.** FA were obtained from adipose tissue. Sampling areas are North-West (NW), North-East/South-West diagonal (NESW) and South-East (SE). Breeding status indicates whereas the female was accompanied or not with cubs and their age: solitary females (Solitary), females with cubs of the year (with COYs) and females with yearlings (with YRLs). Female polar bears were sampled in Svalbard in 2012 and 2013. Bars are median values and standard errors.

Among the selected FA, we only reported results that were significantly different. In females caught in autumn, the proportion of FA c16:0 and 16:1n-7 was higher of 1.41% [0.66; 2.17] (values are parameter estimates and 95% CI) and 2.52% [1.32; 3.72] respectively, compared to females caught in spring (Fig 4A). Additionally, in females caught in autumn we observed a lower proportion of FA 20:1n-9 and 22:1n-9–2.95% [-4.11; -1.78] and -0.13% [-0.20; -0.07] respectively, compared to females caught in spring (Fig 4A). In solitary females, FA 18:1n-9 was lower of -2.92% [-5.25; -0.60] and FA 16:1n-7, 18:4n-3 and 20:5n-3 were higher of 2.73% [0.67; 4.80], 0.31% [0.07; 0.56] and 1.02% [0.18; 1.87] respectively compared to females with COYs (Fig 4B). With regard to the relationships between individual FA variation and sampling area (Table 3), we observed the following pattern: North-West females < NESW, South-East females, for FA 16:1n-7 and 22:5n-3. North-West females > South-East females for FA 18:2n-6, 20:1n-9, 22:1n-9 and 22:1n-11 (Fig 4C). South-East females had higher proportions of c16:0, 16:1n-7 and lower proportions of 20:1n-9, 22:1n-11, 22:1n-9, 22:6n-3 compared to NESW females (Fig 4C).

**Table 3:** Relationships between selected fatty acids (FA) and sampling area.

	North-West—South-East		South-East—NESW		North-West—NESW	
Fatty acids	Estimate	95% CI	Estimate	95% CI	Estimate	95% CI
c16:0	ns	ns	1.42	0.32–2.52	ns	ns
16:1n-7	-0.85	-1.54–-0.15	1.91	0.30–3.54	-0.88	-1.59–-0.17
18:1n-9	ns	ns	ns	ns	ns	ns
18:2n-6	0.21	0.06–0.37	ns	ns	ns	ns
18:4n-3	ns	ns	ns	ns	ns	ns
20:1n-9	2.47	0.44–4.50	-3.00	-4.64–-1.30	ns	ns
20:5n-3	ns	ns	ns	ns	ns	ns
22:1n-11	1.04	0.50–1.58	-0.57	-1.00–-0.14	ns	ns
22:1n-9	0.15	0.04–0.27	-0.12	-0.21–-0.02	ns	ns
22:5n-3	-4.09	-6.06–-2.12	ns	ns	-2.17	-4.18–-0.16
22:6n-3	ns	ns	-1.23	-2.20–-0.26	ns	ns

Females were sampled from Svalbard area in 2012–2013. Values are parameter estimates and 95% confidence intervals (CI) of general linear mixed models that included Female identity as a random factor and sampling area as a fixed factor. “ns” refers to “non-significant” estimates with a 95% CI that crossed zero.

### Predictors of stable isotope values in plasma and red blood cells

**Fig 5:**
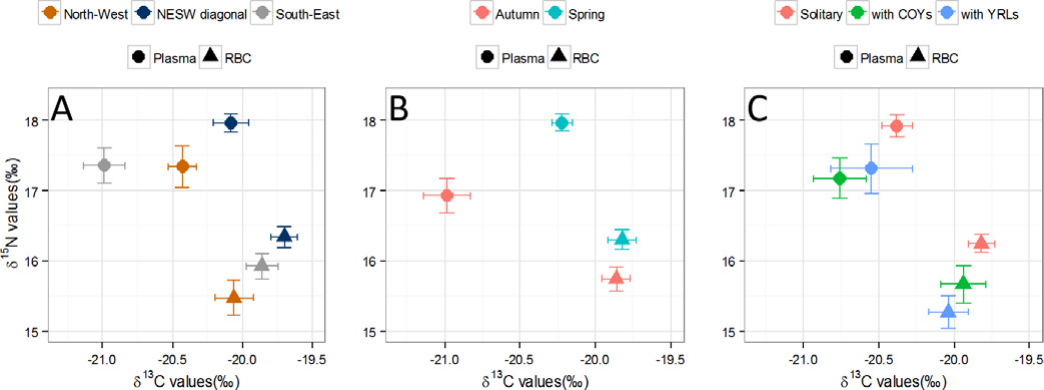
**Relationship between blood stable isotopes and (A) sampling area, (B) season and (C) breeding status.** Nitrogen (δ^15^N) and carbon (δ^13^C) stable isotope values were measured in plasma (circles) and red blood cells (triangles). Seasons are spring (samples from April) and autumn (samples from September). Breeding status indicates whereas the female was accompanied or not with cubs and their age: solitary females (Solitary), females with cubs of the year (with COYs) and females with yearlings (with YRLs). We sampled female polar bears in Svalbard in 2012 and 2013. Circles/triangles represent medians and bars standard error.

Among the variables we included in our models to explain δ^13^C values in red blood cells, the null model (ΔAICc = 1.75) received strong support indicating that little variance in δ^13^C values in red blood cells was explained by the variables we selected (Table 1, Fig 5). In plasma, the best predictors of δ^13^C values were season, sampling area and their interaction (Table 4). The only term that had a significant effect on plasma δ^13^C values was the interaction of season and sampling area (Fig 5). The comparison of autumn δ^13^C values in NESW and South-East females to spring δ^13^C values in North-West females was not biologically relevant. Therefore, we considered the interaction term by testing whether plasma δ^13^C values would seasonally vary in each area. Least squares means method revealed that in the South-East area, females caught in autumn had plasma δ^13^C values lower of 1.22‰ [0.72; 1.71] than in spring. In females caught in the North-West and in the NESW diagonal, we did not observe seasonal differences in plasma δ^13^C values (-0.07‰ [-0.73; 0.58] and 0.21‰ [-0.54; 0.96], respectively).

**Table 4:** Relationships between stable isotope values, environmental factors and breeding status in female polar bears.

Response	δ^13^C values plasma		δ^15^N values red blood cells		δ^15^N values plasma	
Selected model	Sampling area + Season + Sampling area × Season		Sampling area + Status		Season + Status	
	Estimate	95% CI	Estimate	95% CI	Estimate	95% CI
**Fixed Parts**						
Intercept	-20.48	-20.88 - -20.09	15.49	14.89 - 16.10	18.28	17.87 - 18.70
Autumn	0.08	-0.54 - 0.69 ^a^			-0.99	-1.39 - -0.59
NESW	0.32	-0.16 - 0.79 ^a^	1.01	0.33 - 1.69		
South-East	0.16	-0.40 - 0.72 ^a^	0.62	-0.04 - 1.28^a^		
Autumn NESW	-0.28	-1.22 - 0.65 ^a^^,^^b^				
Autumn South-East	-1.30	-2.07 - -0.52 ^b^				
with COYs			-0.30	-0.69 - 0.09 ^a^	-0.41	-0.91 - 0.10 ^a^
with YRLs			-0.66	-1.16 - -0.17	-0.94	-1.56 - -0.31
**Random Parts**						
N_FemID_		78		78		78
ICC_FemID_		0.102		0.721		0.634
Observations		112		112		112

Females were sampled from Svalbard area in 2012–2013. Values are parameter estimates and 95% confidence intervals (CI) for the best general linear mixed models according to AICc selection. All models (linear mixed models) include Female identity (FemID) as a random factor, sampling area, season and breeding status as fixed factors. Parameter estimates represent contrasts to the reference level, which was spring, north-west, and solitary female in 2012. For the random parts, N is the number of individuals, ICC is the intraclass correlation coefficient and Observations the number of samples.

^a^This effect is not supported because the 95% CI included zero.

^b^ This effect will be considered into a biologically relevant data frame as detailed in the results section.

The best predictors of δ^15^N in red blood cells were sampling area and breeding status (Table 4). Females captured in the North-West had δ^15^N values that were 1.01‰ [0.33; 1.69] lower compared to females from the NESW diagonal, yet δ^15^N values did not differ from that of South-East females (Table 4, Fig 5A). Additionally, females with YRLs had δ^15^N values that were lower by 0.66‰ compared to solitary females (Table 4). δ^15^N values were not different between solitary females and those with COYs (Table 4, Fig 5C). In plasma, season and breeding status were significantly related to δ^15^N values (Table 4). In females caught in autumn, plasma δ^15^N values were lower of -0.99‰ [-1.39; -0.59] compared to those caught in spring (Fig 5B). Similarly as in δ^15^N red blood cells values, plasma δ^15^N values in females with YRLs was lower of -0.94‰ [-1.56; -0.31] compared to that of solitary females and we observed no differences between females with COYs plasma δ^15^N values and those of solitary ones (Table 4, Fig 5C).

### Selectivity in diet

For δ^13^C values’ variances measured in plasma we observed the following pattern: South-East > NESW and North-West (South-East, NESW: Levene statistic tests (LST) = 5.1, P = 0.027; South-East, North-West: LST = 7.6, P = 0.008 and North-West, NESW: LST = 0.4, P = 0.517, Fig 5A). For δ^15^N variance the pattern was South-East and North-West > NESW diagonal (South-East, NESW: LST = 12.9, P<0.001; South-East, North-West: LST = 0.6, P = 0.434 and North-West, NESW: LST = 9.6, P = 0.003, Fig 5A). With regard to δ^15^N and δ^13^C values in red blood cells, we did not find any variance difference according to sampling area (LST<3.8, P>0.057 for all tests).

In autumn, the variances of plasma δ^15^N and δ^13^C in female polar bears were larger than that of females caught in spring (plasma δ^15^N: LST = 13.4, P<0.001, plasma δ^13^C: LST = 16.5, P<0.001, for stable isotopes in red blood cells P>0.760 for all tests, Fig 5B). Sampling year was not related to the variances of plasma and red blood cells’ stable isotope values (LST<0.5, P>0.465, for all tests). Finally, the variances of red blood cells’ δ^15^N values in solitary females were smaller than that of females with COYs (red blood cells δ^15^N: LST = 8.0, P = 0.005, Fig 5C); and solitary females had a smaller plasma δ^13^C value variance than females with YRLs (LST = 6.1, P = 0.016, Fig 5C). Specifically, solitary females were more selective (less isotopic variance) than females with COYs and with YRLs, in terms of δ^15^N and δ^13^C values, respectively (Fig 5C). Variances of δ^15^N and δ^13^C values in plasma or red blood cells were not significantly different for any other comparison (P>0.058 for all tests).

## Discussion

Female polar bear habitat area use, life history stage and season are all important drivers of diet based on our isotope and lipid analysis over two years on Svalbard, in the Barents Sea. Previous studies have observed that seal species differ in availability according to locations in Svalbard and distance from the coast [12,61–63] and that in spring female polar bears of different life history stages tend to select different habitats [32,34]. This is the first evidence of such diet variations among female polar bears from the Barents Sea subpopulation. Diet shifts between spring and autumn were less pronounced than predicted. Among females from the South-East we observed a plasma δ^13^C depletion from spring to autumn which could result from the larger amplitude in sea ice extent in that region. Additionally, FA PCA scores and δ^15^N values in red blood cells were lower in fall than spring, which could mirror seasonal differences in trophic level.

### Carbon, nitrogen and lipid sources in relation to sampling area

**Fig 6:**
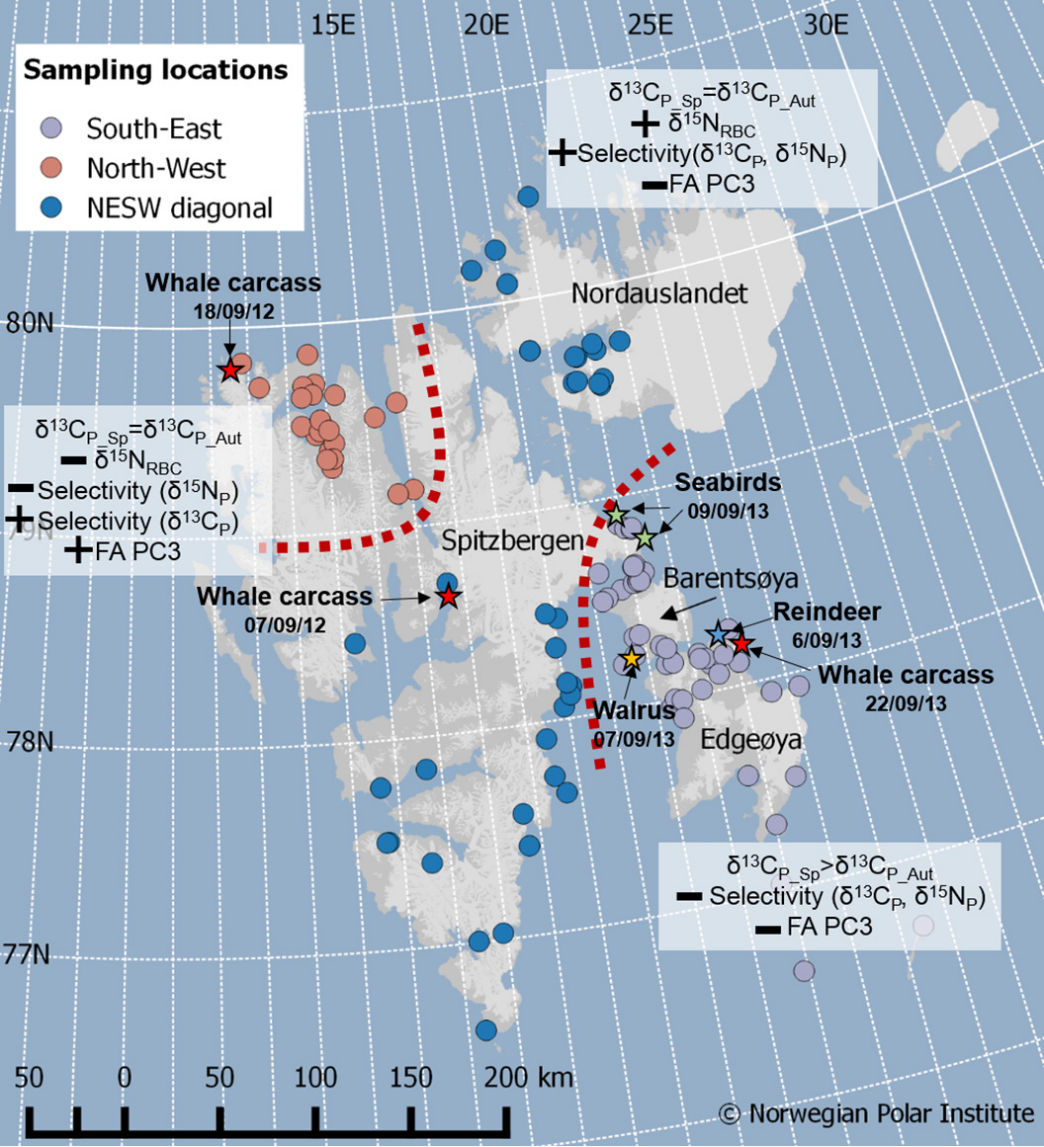
Summary of diet tracer variations according to sampling area. Diet tracers include δ^15^N and δ^13^C values in plasma (e.g δ^13^C_P_) and red blood cells (e.g δ^15^N_RBC_), and fatty acids principal component first axis values (FA PC3). For each area, seasonal differences in plasma δ^13^C values appear as “δ^13^C_P_Sp_ = δ^13^C_P_Aut_”, Sp denotes spring and Aut denotes autumn. Each sign (+ or -) denotes direction in significant differences. For example, δ^15^N_RBC_ values are lower in North-West females compared to NESW females but not different from South-East females. Whereas FA PC3 values are higher in North-West females compared to NESW and South-East females. The red stars indicates the positions of whale carcasses, the orange star indicates the position of a bear feeding on a walrus (*Odobenus rosmarus*) kill, the green stars a bird cliff where bears have been observed feeding on birds and the blue star represents the position of a bear with reindeer hair between the teeth.

As expected, we observed diet variations among sampling areas (**Fig 6**). Since prey distribution may not be uniform in Svalbard [12,61–63], it seems likely that female polar bears in different areas (i.e. North-West, NESW diagonal and South-East) feed on prey species relative to their occurrence. Furthermore, predation on seals will depend on sea ice cover.

The west coast of Spitsbergen typically has less sea ice year round than the eastern part due largely to the relatively warm western Spitsbergen current [26,44,45]. Less sea ice may drive polar bears to feed on prey other than seals to meet their energetic needs. North-West females exhibited lower red blood cell δ^15^N values than females from the NESW diagonal. The FA 22:1n-11 was higher in North-West females and can be used as a marker of pelagic prey [54]. FA 22:1n-11 is higher in fish and seabirds than in seal species or whales [64,65]. Ingesting seabirds and their eggs would likely result in depletion in the δ^15^N values of plasma and red blood cells of the predator, given the lower δ^15^N values of seabirds compared to seals [66]. In Svalbard, δ^15^N value in seabird muscles and eggs (including *Rissa tridactyla*, *Somateria molissima*, *Uria lomvia*, *Alle alle*) averages 11.4 ± 0.4 ‰, whereas in ringed seal muscles, δ^15^N value averages 14.5 ± 0.5 ‰ (E. Fuglei and D. Ehrich unpublished data).

**Fig 7:**
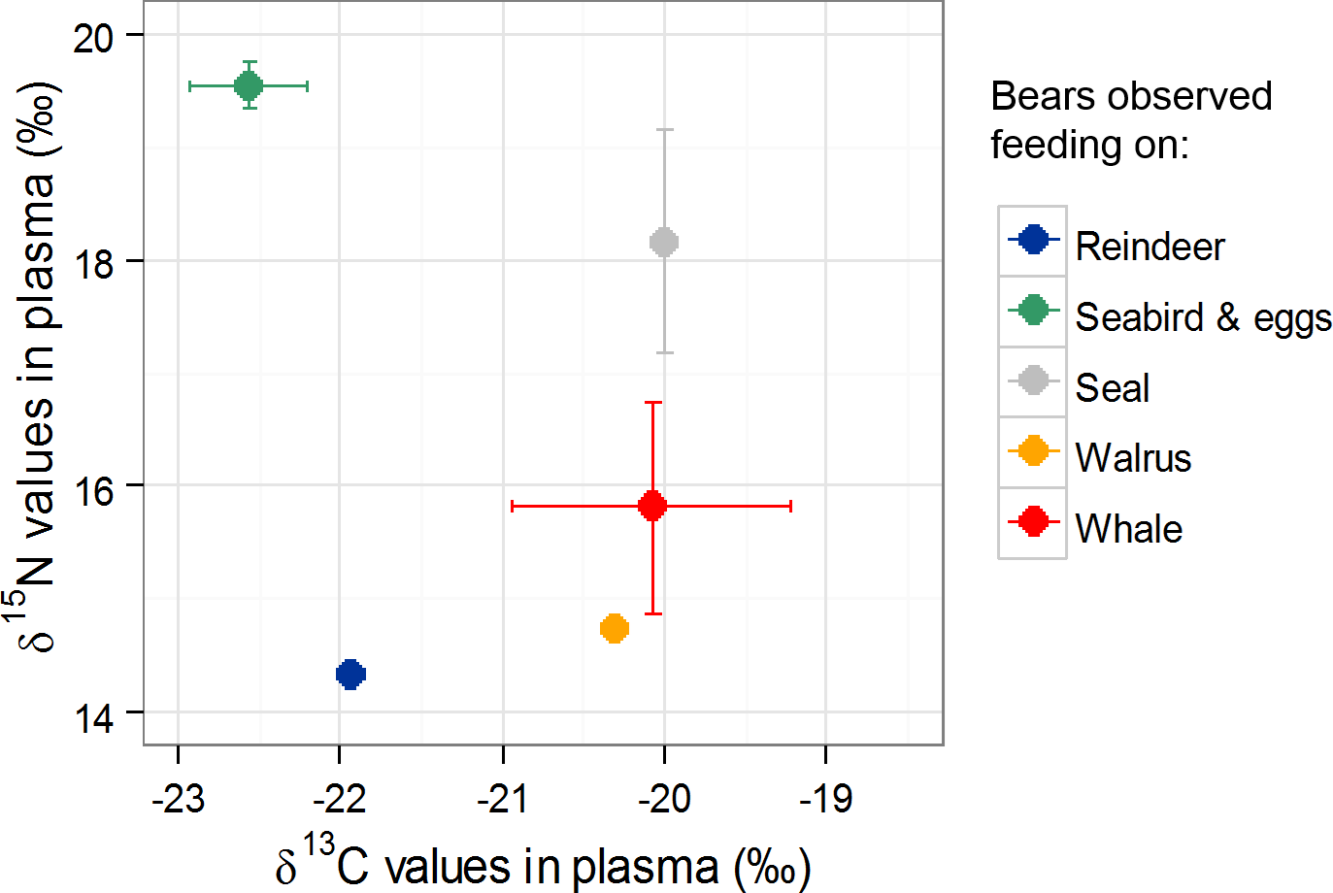
Stable isotopes values measured in plasma of female polar bears observed feeding before capture. Three females with COYs were observed feeding on seabirds (green dot), one female had reindeer hair between the teeth (blue dot), one was observed feeding on a walrus (orange dot), four on three different whale carcasses (red dot) and two on unidentified seals (grey dot).

In females from the South-East, we observed lower plasma δ^13^C values in autumn compared to spring. In comparison to the two other locations, the South-East area experiences the largest amplitude of sea ice retreat during summer [45]. During the ice free season, some females from the South-East area could have been preying on reindeer, seabirds or molting waterfowl and their chicks, as already observed in Svalbard [16,67] which would lead to depleted δ^13^C values in the females. Waterfowl, such as pink-footed goose, barnacle goose or Brent goose (*Anser brachyrhynchus*, *Branta leucopsis*, *Branta bernicla*, respectively) are common in Svalbard [68] and feed on vegetation [69]. δ^13^C values in muscles and eggs of pink-footed and barnacle geese, average -27.3 ± 1.5 ‰, in Svalbard reindeer -26.3 ± 0.6 ‰, while in ringed seals δ^13^C values average -19.8 ± 0.5 ‰ (E. Fuglei and D. Ehrich unpublished data). Additionally, South-East females exhibited high % of 16:1n-7and 16:0. The FA 16:0 is found in high proportions in waterfowl eggs [55,56]. Consequently, δ^13^C values and FA profile in South-East females could integrate a larger proportion of terrestrial food during the ice free season. Indeed, among the females that were captured in 2012 and 2013, some individuals were directly observed preying on seabirds (*R*. *tridactyla*), and one female had reindeer hair between her teeth. The females preying on seabirds and reindeer exhibited depleted δ^13^C values in plasma (Fig 7). Moreover, baleen whales are also expected to have depleted stable isotope values compared to seals [70]. δ^13^C values in plasma obtained from female polar bears feeding on whale carcasses were slightly lower than values from a female that just fed on a seal (Fig 7). Therefore, the fact South-East females could prey on different seal species, whale remains and birds would therefore explain the larger variance in both δ^13^C and δ^15^N stable isotope values in plasma compared to females from the NESW and North-West (δ^13^C values only).

In the NESW diagonal, females seemed to be more selective. This suggests that the access and availability of their preferred prey was higher than in the two other regions. In polar bears, the main feeding period starts early April during the ringed seal pupping peak and lasts until the moulting period of adults in mid- or late June [71–73]. Female ringed seals rely on stable ice conditions to dig out birth lairs and prime breeding areas are found near glacier fronts that terminate at the sea [72,74]. These tidewater glaciers are more numerous in the NESW diagonal than in the North-West or South-East areas [74]. Additionally, in the 1980s, a survey investigated the density of breeding female ringed seals in Svalbard [75]. It appeared that higher densities of birth lairs were found in the western part of Spitsbergen and between Nordaustlandet and Spitsbergen [75]. Consequently, females from the NESW area should have better access to ringed seals compared to the two other locations.

Additionally, the only FA measured in NESW females that was different from both North-West and South-East females, was 16:1n-7 (South-East>NESW>North-West). This FA can be characteristic of body-condition or of differently based food-chain, depicting the access to different type of seal species [57–59]. For example, other seal species could be more accessible in certain locations than others. Hooded seals could be predated by North-West female polar bears, as from October to January they forage in the North of Svalbard [76]. This could in part explain the higher 18:2n-6 and lower 16:1n-7 observed in North-West females as this FA profile is characteristic from the dinoflagellate and the prymnesiophyte based food-chain of hooded seals [58]. In the South-East, polar bears could also prey on harp seals [12]. In June and July, harp seals have been observed in the South-East of Svalbard [77] and their FA signature is derived from a diatom based food chain characterized by higher % of 20:5n-3, 22:5n-3, 16:1n-7 and lower c18 PUFA, and South-East females bore higher % of 22:5n-3, 16:1n-7 and lower 18:2n-6 [57,58].

### Season and diet

For polar bears, predation success in winter is thought to be low [78] then they undergo a hyperphagic period from early April to late June [79]. As previously mentioned, the turnover time of stable isotopes in red blood cells and FA in adipose is likely in the order of weeks to several months while the turnover of stable isotopes in plasma is in the order of 1–2 weeks [36,38,80,81]. Therefore, several blood samples taken in April represent diet at the onset of the hyperphagic period. This may explain the higher δ^15^N values in plasma in comparison to females sampled in autumn and the more specific diet (as depicted by the smaller stable isotopic variances in spring than in autumn). In spring, polar bears will mainly prey on seals, whereas in autumn prey items may be more diversified (e.g. waterfowls and seabirds). Surprisingly, the FA pattern seems less specific in spring than in autumn (Fig 2A). Females were sampled at the beginning of the hyperphagic period, while some of them were still fasting and had not started building energy stores. Indeed, body condition index (BCI) in the same polar bears as used in the present study was lower in spring than in autumn (Bourgeon et al. submitted). When used as an energy source, the release of FA is selective according to carbon chain length and degree of unsaturation (reviewed in [59]). In spring, females had higher proportions of FA 20:1n-9 and 22:1n-9 which are amongst the least mobilized [59]. Conversely, 16:1n-7 is a readily mobilized FA [59] and was in lower proportion in females caught in spring. The higher δ^15^N values measured in females caught in spring could also be the result of a higher proportion of females under nutritional stress. These individuals will catabolize their lean tissues, which will enrich their δ^15^N pool independently of their trophic level [82–84]. Consequently, it is likely that the observed stable isotopic and FA patterns measured in spring resulted from both nutritional stress of some individuals and diet switching.

### Carbon, nitrogen and lipid sources in relation to breeding status

In spring, females of different reproductive status do not use the same habitat [32,34]. Solitary females extensively use pack ice and have a large home ranges, whereas females with COYs mainly remain on fast ice close to glacier fronts [32,34]. In spring, the restriction of females with young cubs to a certain type of habitat could result from habitat selection to reduce the risk of infanticide, reduced mobility and swimming capacities of young cubs, added to a need for access to a predictable food source such as ringed seal pups born in the fast ice habitat [32,34]. Telemetry data indicates that females with YRLs are the reproductive group with the most freedom to move around as they are less limited in their mobility compared to females with COYs and they are not involved in mating [32].

FA profiles in females with YRLs, suggest lipid sources close to that of solitary females. Yet, solitary females have higher δ^15^N values in plasma and red blood cells than females with YRLs. Lactation has been shown to deplete the mother’s δ^15^N pool [85] and female polar bears typically lactate until their cubs are ~1.5 years old [86,87]. Consequently, females with YRLs that have been lactating for more than a year are likely to have lower δ^15^N values in both plasma and red blood cells than solitary females. The FA profile of females with COYs could have resulted from a broader prey base, as evidenced by the larger variance in plasma δ^15^C stable isotope values, or could mirror fasting physiology. Females with COYs sampled in spring have just emerged from their dens and FA in adipose tissues integrate diet information over the previous weeks to the past few months [36]. If some females with COYs have been sampled while fasting, we could expect the observed FA pattern with lower proportions of 16:1n-7 and 20:5n-3 FA [59]. Several fasting females with COYs could explain the observed δ^15^N values (Solitary > with COYs > with YRLs), although lactation is supposed to deplete δ^15^N values in the mother pool, nutritional stress could lead to an increase of δ^15^N values. Moreover, we observed three females with COYs feeding on kittiwakes in autumn 2013 (Fig 5). This could increase the overall higher proportion of 18:1n-9 measured in females with COYs, as this FA is observed to be in high proportion in birds [55,56].

The diet tracer patterns observed in lone females as compared to those with cubs suggest that lone females are the most selective foragers. For example, they have smaller variances in stable isotopes, higher δ^15^N values compared to females with YRLs and different FA composition compared to females with COYs. Lone females are less limited in terms of mobility and nutritional needs (e.g. no lactation) and could thus focus their hunts on higher energy adult ringed seals, thus preparing for future reproduction and growth [88].

In prey-rich environments, large carnivorous mammals are energy maximizers that can afford to select their prey in terms of those species, age, and sex classes that are most abundant or easiest to hunt and kill, whereas in a prey-poor environment predators will maximize the number of prey without selection of species [89,90]. Females with dependent young have to develop additional strategies to increase their own survival and that of their offspring [90]. This is what we observe in female polar bears and similar strategies have been observed in other carnivorous mammals. For example, in leopards (*Panthera pardus*), cheetahs (*Acinonyx jubatus*) and Eurasian otters (*Lutra lutra*), females with young tend to forage in different areas than solitary females and males, take fewer risks of encountering potential predators and be less selective on prey [90–93].

## Conclusion and Perspectives

This study provides new understanding of the ecological factors contributing to the diet of female polar bears in Svalbard and our data show diet specialization over a small geographic scale. Polar bears’ movements follow a circannual pattern with season-specific area fidelity [94–96]. Considering this, even within a small portion of the Barents Sea polar bear subpopulation, some individuals may be more threatened by sea ice retreat according to their season-specific foraging area. In the North-West females have lower BCI than in other areas (Bourgeon et al. submitted), whereas in the South-East females experience the largest amplitude in seasonal sea ice retreat. We can postulate that females from those two areas may be more sensitive to climate changes than NESW females, which have a more consistent habitat and thus a more consistent prey availability. Our findings suggest that complex changes in sea ice, prey availability and life history status could all interact to determine female polar bear feeding patterns in Svalbard.

## Supporting Information

S1 Appendix Fatty acid and stable isotope determination. (DOCX)

S1 Table Number of samples available for stable isotopes in plasma and red blood cells (n = 112) and fatty acids in adipose tissue (n = 83) of female polar bears from Svalbard (2012–2013). Samples are then sorted by year, sampling location, breeding status and season.
(DOCX)

S2 Table Average (± standard deviation, SD) mass % of fatty acids in polar bear adipose tissue. Samples were collected from 83 females in Svalbard archipelago in 2012 and 2013. FA in bold are those selected for statistical analyses.
(DOCX)

S3 Table List of the 15 candidate models used for model selection. We used model selection via AICc to determine the best predictors of nitrogen and carbon stable isotope values in plasma and red blood cells, and fatty acid composition in adipose tissue (using principal components values).
(DOCX)
