# Supplementary material for: Geographical Area and Life History Traits Influence Diet in an Arctic Marine Predator
Source: PLoS One. 2016 May 19;11(5):e0155980. doi: 10.1371/journal.pone.0155980 (PMC4873193; doi:10.1371/journal.pone.0155980)
Supplement: S1 Appendix — (DOCX) [file pone.0155980.s001.docx]

**S1 APPENDIX**

**Fatty acid determination in adipose tissue**

Lipid was quantitatively extracted from each adipose tissue sample according to [1] and FA methyl esters (FAME) were prepared using H_2_SO_4_ as a catalyst [2]. Duplicate analyses and identification of FAME were performed using temperature-programmed gas–liquid chromatography according to [3] and [4,5]. Samples were analyzed on a Perkin Elmer Autosystem II Capillary gas chromatograph with a flame ionization detector fitted with a flexible fused silica column (30 m × 0.25 mm inner diameter) coated with 50% cyanopropyl polysiloxane (0.25-μm film thickness) (DB-23; Agilent Technologies, Palo Alto, California, USA). FA data are expressed as the mass percentage of total FA ± 1 standard error of the mean (SEM). Individual FA are referred to by the shorthand nomenclature of carbon-chain length:number of double bonds, and position of the first double bond relative to the terminal methyl group.

1. Iverson SJ, Lang SL, Cooper MH. Comparison of the Bligh and Dyer and Folch methods for total lipid determination in a broad range of marine tissue. Lipids. 2001;36: 1283–1287.

2. Thiemann GW, Budge SM, Iverson SJ. Determining blubber fatty acid composition: a comparison of in situ direct and traditional methods. Mar Mammal Sci. 2004;20: 284–295.

3. Iverson SJ, Frost KJ, Lang L. Fat content and fatty acid composition of forage fish and invertebrates in Prince William Sound, Alaska: factors contributing to among and within species variability. Mar Ecol Prog Ser. 2002;241: 161–181.

4. Budge SM, Iverson SJ, Bowen WD, Ackman RG. Among-and within-species variability in fatty acid signatures of marine fish and invertebrates on the Scotian Shelf, Georges Bank, and southern Gulf of St. Lawrence. Can J Fish Aquat Sci. 2002;59: 886–898.

5. Budge SM, Iverson SJ, Koopman HN. Studying Trophic Ecology in Marine Ecosystems Using Fatty Acids: A Primer on Analysis and Interpretation. Mar Mammal Sci. 2006;22: 759–801. doi:10.1111/j.1748-7692.2006.00079.x

**Stable isotopes determination in plasma and red blood cells**

We dried plasma and red blood cells at 50°C for 3 days and homogenized the samples using a bead-mill homogenizer (TissueLyzerII, Qiagen Gmbh, Hilden, Germany). We weighed all samples on a Sartorius ME5 microbalance (Sartorius AG, Goettingen, Germany) and packed all samples in 3.5 × 5 mm tin cups for analysis. We analyzed the samples using a Costech ECS 4010 elemental analyzer (Costech, Valencia, CA) in line with a ThermoFinnigan DeltaPlus XP continuous-flow isotope ratio mass spectrometer (CF-IRMS; Thermo Scientific, Bremen, Germany) for δ^13^C and δ^15^N, which was calibrated against international reference standards from the International Atomic Energy Agency (IAEA-N1, IAEA-CH7, IAEA-C3, and IAEA-600) and the USGS (USGS-25, USGS-40, and USGS-41). We included internal standards of purified methionine (Alfa Aesar, δ^13^C = −34.58 ± 0.06 ‰, δ^15^N = −0.94 ± 0.16 ‰; all error data are SD) and homogenized Chinook salmon muscle (UAA Stable Isotope Lab, δ^13^C = −19.27 ± 0.05 ‰, δ^15^N = 15.56 ± 0.13 ‰) with all samples as quality controls. SI values are reported in standard δ notation and are referenced to Vienna Pee Dee Belemnite (VPDB) for δ^13^C and to air for δ^15^N. Long-term records of internal standards yield an analytical precision of 0.12 ‰ for δ^15^N and 0.11 ‰ for δ^13^C. Replicates were analyzed to determine intra-individual variability. We conducted solids analysis at the Environment and Natural Resources Institute Stable Isotope Laboratory at the University of Alaska, Anchorage (http://​www.​uaa.​alaska.​edu/​enri/​labs/​sils). Quality assurance is based on the standard uncertainty of the known value of the reference material, three purified CO_2_ gas aliquots (INSTAAR, University of Colorado, δ^13^C = −3.32, −10.04 and −24.68 ‰). If the standard uncertainty is larger than 0.3 ‰, the samples were re-analyzed (until the 2-sigma expanded standard uncertainty of the result is <0.6 ‰).
