# Supplementary material for: Geographical Area and Life History Traits Influence Diet in an Arctic Marine Predator
Source: PLoS One. 2016 May 19;11(5):e0155980. doi: 10.1371/journal.pone.0155980 (PMC4873193; doi:10.1371/journal.pone.0155980)
Supplement: S1 Table — Samples are then sorted by year, sampling location, breeding status and season. (DOCX) [file pone.0155980.s002.docx]

**S1 Table.**

**Number of samples available for stable isotopes in plasma and red blood cells (n=112) and fatty acids in adipose tissue (n=83) of female polar bears from Svalbard (2012-2013).**

|  |  | Stable isotopes in blood | FA in adipose tissue |
| --- | --- | --- | --- |
|  |  | # samples | # samples |
| Total | | **112** | **83** |
| Year | 2012 | 57 | 40 |
|  | 2013 | 55 | 43 |
| Sampling location | North-West | 24 | 18 |
|  | NESW | 38 | 30 |
|  | South-East | 50 | 35 |
| Breeding status | Solitary | 55 | 47 |
|  | with COYs | 34 | 16 |
|  | with YRLs | 23 | 20 |
| Season | Spring | 62 | 48 |
|  | Autumn | 50 | 35 |

Samples are then sorted by year, sampling location, breeding status and season.
