# Supplementary material for: Geographical Area and Life History Traits Influence Diet in an Arctic Marine Predator
Source: PLoS One. 2016 May 19;11(5):e0155980. doi: 10.1371/journal.pone.0155980 (PMC4873193; doi:10.1371/journal.pone.0155980)
Supplement: S2 Table — Samples were collected from 83 females in Svalbard archipelago in 2012 and 2013. FA in bold are those selected for statistical analyses. (DOCX) [file pone.0155980.s003.docx]

**S2 Table.**

**Average (± standard deviation, SD) mass % of fatty acids in polar bear adipose tissue.**

| Fatty acids | Mean ± SE |  | Fatty acids | Mean ± SE |  | Fatty acids | Mean ± SE |
| --- | --- | --- | --- | --- | --- | --- | --- |
| c12:0 | 0.01 ± 0.01 |  | 16:3n-4 | 0.10 ± 0.10 |  | 20:2n-9 | 0.02 ± 0.03 |
| BHT | <0.01 |  | c17:1 | 0.04 ± 0.03 |  | 20:2NMID2 | 0.04 ± 0.03 |
| c13:0 | 0.01 ± 0.01 |  | 16:4n-3 | 0.06 ± 0.06 |  | **20:2n-6** | 0.36 ± 0.09 |
| **i-14:0** | 0.66 ± 1.23 |  | 16:4n-1 | 0.02 ± 0.01 |  | 20:3NMIT | 0.05 ± 0.02 |
| **c14:0** | 2.06 ± 1.21 |  | **c18:0** | 2.77 ± 1.28 |  | 20:3n-6 | 0.15 ± 0.03 |
| 14:1n-9 | 0.04 ± 0.02 |  | 18:1n-13 | 0.06 ± 0.03 |  | **20:4n-6** | 0.36 ± 0.14 |
| 14:1n-7 | 0.17 ± 0.27 |  | **18:1n-11** | 2.43 ± 1.17 |  | 20:3n-3 | 0.08 ± 0.02 |
| **14:1n-5** | 0.52 ± 0.21 |  | **18:1n-9** | 25.27 ± 3.35 |  | **20:4n-3** | 0.65 ± 0.17 |
| i-15:0 | 0.19 ± 0.08 |  | **18:1n-7** | 4.81 ± 0.85 |  | **20:5n-3** | 2.62 ± 1.22 |
| ai-15:0 | 0.07 ± 0.04 |  | **18:1n-5** | 0.41 ± 0.06 |  | c22:0 | 0.01 ± 0.01 |
| **c15:0** | 0.25 ± 0.05 |  | 18:2d5,11 | 0.06 ± 0.01 |  | **22:1n-11** | 1.40 ± 0.76 |
| i-16:0 | 0.14 ± 0.03 |  | 18:2n-7 | 0.09 ± 0.02 |  | **22:1n-9** | 0.45 ± 0.16 |
| **c16:0** | 7.96 ± 1.80 |  | **18:2n-6** | 1.81 ± 0.20 |  | 22:1n-7 | 0.08 ± 0.04 |
| **16:1n-11** | 0.23 ± 0.08 |  | 18:2n-4 | 0.09 ± 0.03 |  | 22:2NMID1 | 0.05 ± 0.04 |
| **16:1n-9** | 0.54 ± 0.08 |  | 18:3n-6 | 0.10 ± 0.03 |  | 22:2NMID2 | 0.05 ± 0.03 |
| **16:1n-7** | 11.11 ± 3.00 |  | **18:3n-4** | 0.20 ± 0.03 |  | 22:3NMIT | 0.05 ± 0.03 |
| **16:1n-5** | 0.22 ± 0.05 |  | **18:3n-3** | 0.63 ± 0.15 |  | **21:5n-3** | 0.50 ± 0.10 |
| 17:1(a) | 0.03 ± 0.01 |  | 18:3n-1 | 0.05 ± 0.02 |  | c23:0 | 0.02 ± 0.03 |
| **i-17:0** | 0.21 ± 0.05 |  | **18:4n-3** | 0.74 ± 0.35 |  | 22:4n-6 | 0.18 ± 0.09 |
| 16:2n-6 | 0.03 ± 0.01 |  | 18:4n-1 | 0.13 ± 0.05 |  | 22:5n-6 | 0.16 ± 0.07 |
| ai-17:0 | 0.18 ± 0.07 |  | c20:0 | 0.07 ± 0.04 |  | 22:4n-3 | 0.15 ± 0.09 |
| 17:1(b) | 0.13 ± 0.04 |  | **20:1n-11** | 2.22 ± 0.78 |  | **22:5n-3** | 5.55 ± 0.93 |
| **16:2n-4** | 0.38 ± 0.11 |  | **20:1n-9** | 10.19 ± 2.95 |  | c24:0 | 0.01 ± 0.01 |
| **c17:0** | 0.23 ± 0.08 |  | **20:1n-7** | 0.61 ± 0.38 |  | **22:6n-3** | 8.60 ± 1.64 |
| Phytane | <0.01 |  | 20:2NMID1 | 0.05 ± 0.04 |  | c24:1 | 0.07 ± 0.04 |

Samples were collected from 83 females in Svalbard archipelago in 2012 and 2013. FA in bold are those selected for statistical analyses.
