# Supplementary material for: Geographical Area and Life History Traits Influence Diet in an Arctic Marine Predator
Source: PLoS One. 2016 May 19;11(5):e0155980. doi: 10.1371/journal.pone.0155980 (PMC4873193; doi:10.1371/journal.pone.0155980)
Supplement: S3 Table — We used model selection via AICc to determine the best predictors of nitrogen and carbon stable isotope values in plasma and red blood cells, and fatty acid composition in adipose tissue (using principal components values). (DOCX) [file pone.0155980.s004.docx]

**S3 Table**

**List of the 15 candidate models used for model selection.**

| **Candidate models to explain diet tracers** |
| --- |
| Sampling area |
| Season |
| Status |
| Year |
| Sampling area + Season |
| Sampling area + Status |
| Sampling area + Year |
| Sampling area + Season + Sampling area × Season |
| Sampling area + Status+ Sampling area × Status |
| Sampling area + Year+ Sampling area × Year |
| Season + Status |
| Season + Status + Season × Status |
| Year + Status + Year × Status |
| Year + Season + Status + Season × Status + Year × Season |
| Null model |

We used model selection via AICc to determine the best predictors of nitrogen and carbon stable isotope values in plasma and red blood cells, and fatty acid composition in adipose tissue (using principal components values).
